# Hereditary gingival fibromatosis: Characteristics and treatment approach

**DOI:** 10.4317/jced.53644

**Published:** 2017-04-01

**Authors:** Pedro J. Almiñana-Pastor, Pedro J. Buitrago-Vera, Francisco M. Alpiste-Illueca, Montserrat Catalá-Pizarro

**Affiliations:** 1 DD, Post-graduated in Periodontics, Department d´Estomatologia, Facultad de Medicina y Odontologia, Universidad de Valencia, Valencia, Spain; 2 MD DD, PhD in Medicine. Adjunct Professor of Periodontics, Facultad de Medicina y Odontologia, Universidad de Valencia, Valencia, Spain; 3 MD DD, PhD in Medicine. Assistant Professor of Periodontics, Department d´Estomatologia, Facultad de Medicina y Odontologia, Universidad de Valencia, Valencia, Spain; 4 MD DD, PhD in Medicine. Associate Professor of Pediatric Dentistry, Department d´Estomatologia, Facultad de Medicina y Odontologia, Universidad de Valencia, Valencia, Spain

## Abstract

Hereditary gingival fibromatosis (HGF) is a rare disorder characterized by a benign, non-hemorrhagic, fibrous gingival overgrowth that can appear in isolation or as part of a syndrome. Clinically, a pink gingiva with marked stippling can be seen to cover almost all the tooth, in many cases preventing eruption. HGF usually begins during the transition from primary to permanent teeth, giving rise to a condition that can have negative psychological effects at that age. As it does not resolve spontaneously, the treatment of choice is gingivectomy, which can be performed with an internal or external bevel incision, depending on each case and bearing in mind the changes that will take place at the dentogingival junction (DGJ). This paper describes clinical aspects and treatment in two eight-year-old boys with HGF, considering different facets of the surgical approach with conscious sedation in young children.

** Key words:**Hereditary gingival fibromatosis, gingivectomy, internal bevel incision, external bevel incision, gingival overgrowth.

## Introduction

Hereditary gingival fibromatosis (HGF) is a benign, non-hemorrhagic, fibrous gingival overgrowth that can cover all or part of the teeth. In the current periodontal diseases and conditions classification developed by Armitage in 1999, HGF is one of the categories of Gingival lesions of genetic origin in the Gingival Diseases section (1-5). The prevalence of this condition is low (1/175,000 inhabitants), but a number of cases may occur within the same family. HGF can appear on its own or as part of a syndrome (2,5). It presents an autosomal dominant inheritance pattern although its penetrance and expressivity are variable. Indeed, 20% of cases appear with no family history of the condition(6). The main differential diagnosis is with drug-induced gingival hyperplasia; the drugs cited include phenytoin, cyclosporine and nifedipine (7).

Concerning the etiopathogeny of HGF, the mechanism that leads to gingival enlargement is unknown. Authors have agreed that there is more sub-epithelial fibroblast proliferation and greater collagen and fibronectin synthesis and, at the same time, a reduction in the matrix metalloproteinases (MMPs) entrusted with collagen degradation (4,8-12).

HGF normally appears with the eruption of the permanent teeth, although cases have been described in primary teeth and even at birth (3). It would appear that the inflammatory stimulus of eruption raises the transforming growth factor ß1 (TGFß1) levels (3,5,10-12). The rise in TGFß1 leads to increased proliferation of fibroblasts, which remain phenotypically activated synthesizing collagen and fibronectin. This would cause an imbalance between the synthesis and degradation of extracellular matrix molecules (3,5,8-12).

The HGF gingiva has a rosy color, a fibrous appearance, and marked stippling, but no sign of inflammation. It covers the teeth partially or totally and can be localized or generalized, with a variable degree of severity, but does not affect the bone (2,4,5,8,11). It usually interferes with speech, lip closure and chewing but above all, at the ages at which it appears, it can become a psychological burden and affect the patient’s self-esteem (2,5).

HGF does not resolve spontaneously and the treatment of choice is gingivectomy, which can be performed with an internal or external bevel incision (2,5). Performing surgery after eruption of the permanent teeth reduces the rate of recurrence (2). However, the negative psychological effect of HGF on the patient, and the functional difficulties it can cause, justify running the risk of recurrence and performing surgery at earlier ages.

This article presents two cases, focusing on the clinical characteristics of each and the treatment approach employed.

## Case Reports

-Case 1. A five year-old boy was referred to the Pediatric Dentistry Clinic due to absent front teeth and the extremely swollen appearance of the gingiva, which prevented him from closing his lips. The patient had no relevant medical history, although his maternal uncle had gingival enlargement.

**Figure 1:**
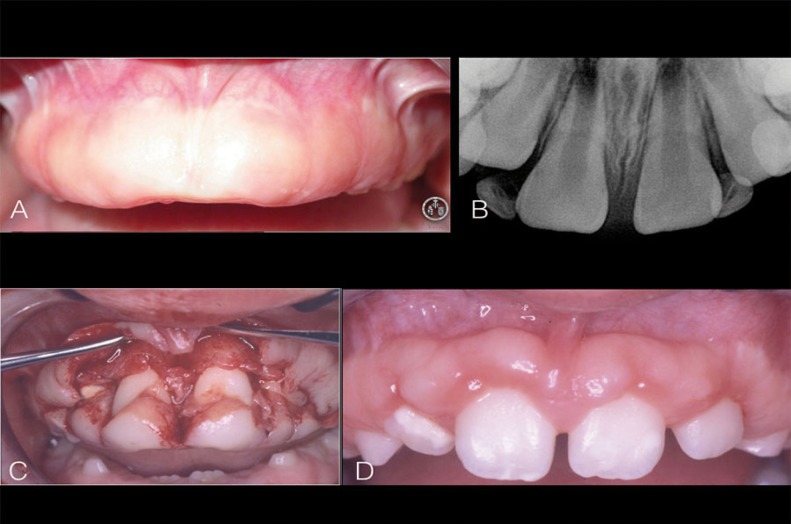
Case 1: A) intraoral view; B) Occlusal radiograph; C) Intrasurgical view of infernal bedel incisión gingivectomy prior to eliminación of excess tissue; D) Intraoral view 6 months after surgery.

At 8 years of age, the permanent teeth had not yet started to erupt and the overgrowth was causing social interaction problems (Fig. 1A). Radiography showed appropriate root development, with two thirds of the root formed (Fig. 1B). At that point it was suggested to the parents that the existing dysfunction should be solved surgically, with nitrous oxide sedation and local anesthesia. The technique chosen by the team of pediatric dentists and periodontists was internal bevel gingivectomy with a full-thickness flap, allowing the tissues to be placed more apically at an appropriate distance from the bone crest. The surgery uncovered the eight permanent incisors, restoring the smile, appearance and function typical of an eight-year-old (Fig. 1C). The child was monitored with regular follow-up visits every three months for 15 months, after which he moved to another town and follow-up was lost (Fig. 1D).

**Figure 2:**
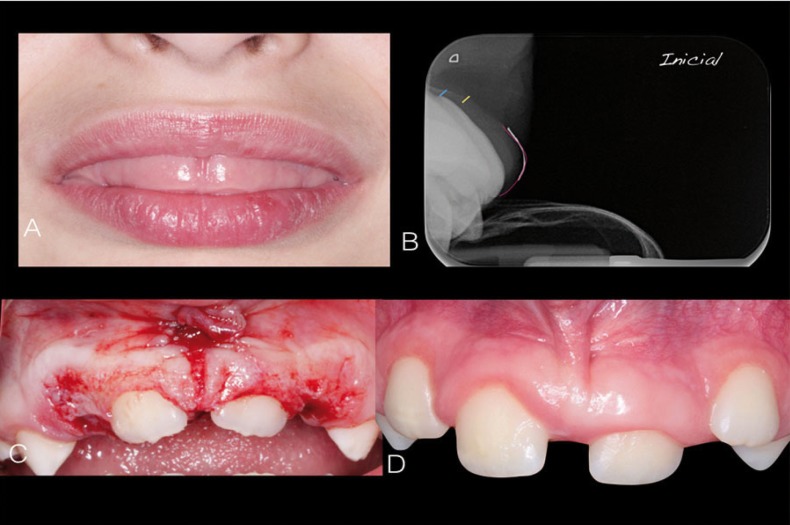
Case 2: A) Lack of lip closure; B) Parallel profile radiograph showing gingiva and bone crest relation; C) Intrasurgical view of external bevel incision gingivectomy; D) Intraoral view 24 months after surgery.

-Case 2. An eight year-old boy was referred due to lack of eruption of front teeth and the swollen appearance of the gingiva, which prevented him from closing his lips (Fig. 2A). The patient had no medical history of interest, although his father and paternal grandfather had had gingival fibromatosis. Clinical examination showed persistence of the primary incisors, no eruption of the permanent incisors and lack of lip closure. Radiography revealed that two thirds of the roots were already formed, indicating that clinical eruption should have taken place. A specific x-ray view (13) showed the proximity of the cemento-enamel line to the bone crest, as well as the disproportionate thickness of the soft tissues (Fig. 2B). The patient was treated by a team of pediatric dentists and periodontists. It was suggested to the parents that surgery should be performed with nitrous oxide sedation and local anesthesia, opting for external bevel gingivectomy. Surgery uncovered the two permanent central incisors, restoring a more appropriate appearance for an eight-year-old boy and favorable periodontal conditions for maintenance and development of the dentogingival junction, which was at a transitional stage of eruption (Fig. 2C). Fifteen months later, with regular follow-up visits every three months, the patient showed eruption progress, possibly encouraged by the gingival tissue removal (Fig. 2D).

**Figure 3:**
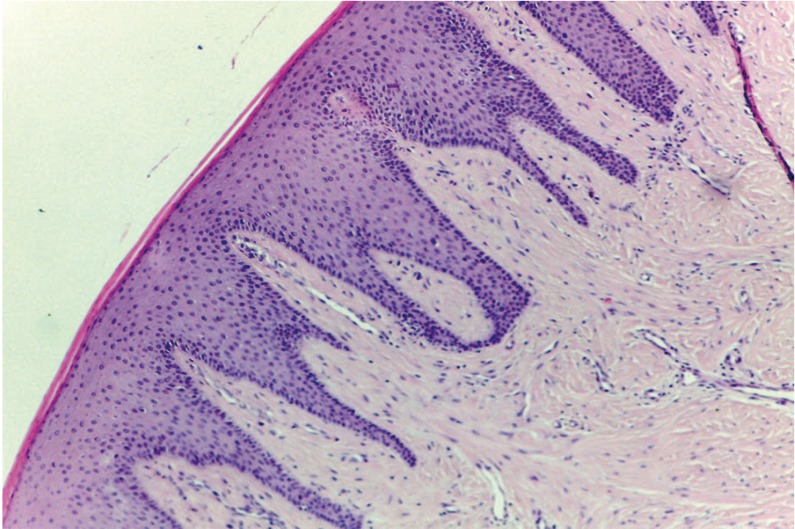
Histological image of Case 2.

The histological images showed acanthosis of the epithelium with fine, elongated epithelial ridges that reached down into dense, highly-stained connective tissue (Fig. 3).

## Discussion

When HGF is suspected, the first step is to rule out medication or a medical or systemic history that could explain the gingival enlargement. A similar history in other members of the family points to a hereditary etiology but is not an exclusive diagnostic criterion for HGF, so the anatomopathologic diagnosis and the progress of the case are indispensable for establishing this diagnosis.

In the two cases reported here, the histological images confirmed the features that characterize HGF (Fig. 3). Nowadays, owing to high genetic heterogeneity, genetic testing to confirm the diagnosis is not justified (14).

The treatment of choice continues to be internal or external bevel gingivectomy. The surgical technique employed has not been shown to influence the risk of recurrence. Lacking more specific criteria in the literature, the choice appears to depend on age, stage of eruption, and the surgeon’s preferences. A cold scalpel provides greater tactile sensitivity and is preferable to laser: since there is no certainty about where the different components of the periodontium are located, there is a risk of excessive destruction of the soft tissues, root tissue or even the bone through direct removal or secondary thermal effects (15).

In the two cases presented here, the DGJ was going through the physiological process of tooth eruption. In the first case, internal bevel gingivectomy made it possible to view the bone crest and its relationship with the cemento-enamel line. However, this technique lengthened the operation time considerably. In the second case, the external bevel technique allowed the gingivectomy and gingivoplasty to be performed without raising a flap, avoiding the need for suture, and was more conservative towards the developing DGJ. In both cases it was found that on eliminating the gingival fibrosis, eruption proceeded physiologically.

When selecting the surgical technique, the following should be considered.

A) Age of the patient: in children or not very collaborative patients, the external bevel technique is faster and does not require suture. B) Stage of eruption: if eruption is complete, the internal bevel is preferable as it allows the tissues to be placed apically after raising a flap and makes it possible to perform osteoplasty if necessary. The external bevel technique is more conservative towards the DGJ during the mixed dentition period.

Although the parents should be warned about the high risk of recurrence, the aesthetic, psychological and functional improvement justifies performing gingivectomy as soon as sufficient root development has been confirmed.
